# Accidental *Ascaridia nymphii* Infection Causing Gastrointestinal Impaction and Hepatic Migration in a Domestic Pigeon in California

**DOI:** 10.3390/ani16101464

**Published:** 2026-05-10

**Authors:** Carlos Daniel Gornatti-Churria, Carmen F. Jerry, Heather M. Fritz, Simone T. Stoute

**Affiliations:** 1 Unidad Académica de Avicultura, Facultad de Veterinaria, Universidad de la República, Montevideo 13000, Uruguay; 2 California Animal Health and Food Safety Laboratory System (CAHFS), Turlock Branch, School of Veterinary Medicine, University of California, Davis, CA 95380, USA; cfjerry@ucdavis.edu (C.F.J.); ststoute@ucdavis.edu (S.T.S.); 3 California Animal Health & Food Safety Laboratory System, Davis Branch, School of Veterinary Medicine, University of California, Davis, CA 95616, USA; hmfritz@ucdavis.edu

**Keywords:** accidental infection, *Ascaridia nymphii*, ascaridiosis, California, liver, pigeon

## Abstract

*Ascaridia nymphii* is a roundworm species affecting domestic avian species, initially described in 2015. One pen-reared, 4-year-old, female American Show Racer pigeon (*Columba livia* f. *domestica*) was submitted to the California Animal Health and Food Safety Laboratory System (CAHFS) Turlock branch, University of California–Davis, for postmortem examination and diagnostic work-up. Grossly, large numbers of ascarids were in the lumen of the proventriculus, gizzard, and duodenum, and a small number was present in the lumen of the trachea, esophagus, and crop. A focal, coiled adult nematode was embedded in the hepatic parenchyma. Ascarids were tan and measured approximately 3.5–4.5 cm in length. The liver was moderately enlarged, green-tinged, and had small, firm, and off-white scattered nodules. Microscopically, we observed multifocal to coalescing granulomas containing intralesional nematodes delineated by necrotic debris, multinucleated giant cells, eosinophilic and heterophilic inflammation, hemorrhage, and bacterial colonies in the liver. The genotypic characterization of the *Ascaridia* sp. in our case (GenBank database accession PX488893) shared 100% identity with *A. nymphii* isolated from the intestinal tract of a cockatiel (*Nymphicus hollandicus*) from Japan in 2015 (GenBank database accession LC057210.1) based on PCR and sequence analysis of an 815 bp segment of the 18S rRNA gene. This report describes the accidental *A. nymphii* infection, which caused severe gastrointestinal impaction and hepatic migration in a domestic pigeon.

## Simple Summary

Gastrointestinal ascaridiosis is a parasitic disease well-known in domestic and wild pigeons. While *Ascaridia columbae* and *Ascaridia galli* infections have been reported as the ascarid species most commonly found in Columbiformes, there are few reports of *Ascaridia nymphii* intestinal parasitemia in Columbiformes. *A. nymphii* has also been reported in psittaciform birds, affecting the gastrointestinal system. Here, we presented an accidental *A. nymphii* infection causing gastrointestinal impaction and hepatic migration in a domestic pigeon (*Columba livia* f. *domestica*) submitted for postmortem examination and laboratory diagnostic work-up.

## 1. Background

Gastrointestinal ascaridiosis is a well-documented parasitic disease that impacts the health and welfare of domestic (*Columba livia* f. *domestica*) and wild pigeons globally [1,2,3,4,5,6,7,8,9,10,11]. In Columbidae, a mild ascarid infection of the gastrointestinal tract leads to weight loss, poor growth, and poor racing performance, while a heavy infection can induce vomiting, diarrhea, emaciation, and mortality [4,6,9,12]. *Ascaridia columbae* and *Ascaridia galli* are considered the most common ascarid species in Columbidae; they parasitize the digestive system with and without liver-lung migration, respectively [1,2,3,4,5,6,8,9,10]. Accidental ascarid infection happens when non-typical avian hosts are affected [6]. In 2015, the species *Ascaridia nymphii* was described in a pet cockatiel (*Nymphicus hollandicus*) in Japan [12]. The *A. nymphii* recovered from the alimentary tract of the cockatiel was classified and differentiated from other ascarid species based on key morphological characteristics and confirmed by molecular identification using sequence analysis of the 18S rRNA gene and NADH dehydrogenase subunit 2 gene (*nad2*) [12]. Subsequent reports of *A. nymphii* were described from fecal samples from three zoo-housed green-winged macaws (*Ara chloroptera*) in China [13] and from fecal samples from domestic pigeons in Iraq [5] and China [14].

The aim of our work is to describe the postmortem diagnostic findings that led to the morphologic and genotypic identification of an accidental *A. nymphii* infection, which led to digestive parasitism and hepatic migration in an American Show Racer pigeon in California.

## 2. Case Presentation

One live, pen-reared, 4-year-old, female American Show Racer pigeon was submitted to the California Animal Health and Food Safety Laboratory System (CAHFS) Turlock branch (University of California–Davis) for postmortem examination and diagnostic work-up. The pigeon was submitted from a flock of 50 multi-age domestic pigeons from an avian hobbyist farm in the Central Valley, California. Birds were housed in a prefabricated shed with solid flooring covered with pine shavings, which was cleaned and replaced monthly. The owner reported challenges with wild birds intermittently accessing the pigeon housing, as well as issues with caking and rapid fecal accumulation in the bedding. Reported flock morbidity was 40% (20/50), and mortality was 24% (12/50) at the time of the lab submission. Two weeks before the lab submission, the owner observed anorexia, lethargy, caking of dried feces on vent feathers, and poor caudal plumage in the affected pigeon. The owner administered a molasses-based nutrition supplement (Poultry Nutri-Drench supplement, Bovidr Laboratories), praziquantel 18 mg/mL, and moxidectin 1 mg/mL dewormer (5 mL MoxiVet Plus (Vetafarm)/19 L drinking water) to the studied bird and VetRx Poultry Remedy supplement for 2 days, but no improvement was observed.

**Figure 1:**
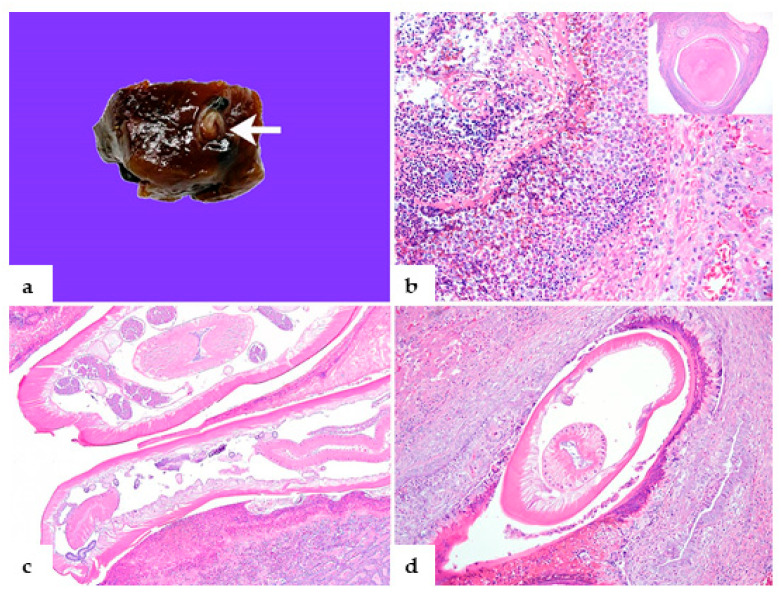
(**a**–**d**) Sections of the liver (**a**,**b**,**d**) and ventriculus (**c**) collected at the necropsy from a pigeon with an *Ascaridia nymphii* accidental infection. (**a**) Thawed section of the fresh, moderately enlarged, green-tinged liver with small, firm, and tan scattered nodules, showing the coiled adult nematode embedded within the parenchyma (white arrow). (**b**) An ascarid hepatic migration track with hemorrhage, eosinophilic infiltrates, and fibrin centrally rimmed by fibroblasts and lymphocytes; the inset shows the chronic migration tract with the caseous material centrally rimmed by the fibrous connective tissue and multinucleated giant cells (granuloma). (**c**) Ventriculus with the adult nematodes, scarce ova, and necrotizing eosinophilic ventriculitis. (**d**) Adult nematode within the migration track surrounded by necrotic debris, multinucleated giant cells, and mixed inflammation in the liver.

On antemortem examination, we noted fair body condition, depression, accumulation of caked feces on the feathers surrounding the vent, and poor caudal plumage. The pigeon was euthanized with CO_2_ gas, and blood was collected from the femoral vein to obtain serum for routine serologic tests, according to standard procedures of CAHFS and following the American Veterinary Medical Association (AVMA) guidelines. Specifically, we performed agar gel immunodiffusion for avian influenza A virus (avian IAV) serology and hemagglutination inhibition tests for detection of *Mycoplasmoides* (*Mycoplasma*) *gallisepticum* (MG), *Mycoplasmopsis* (*Mycoplasma) synoviae* (MS) and avian paramyxovirus types 1, 2, and 3 (avian PMV-1, 2, 3) antibodies. We performed microagglutination testing for *Salmonella* Pullorum and *Salmonella* Gallinarum antibody screening. We prepared blood smears and stained them with Diff-Quick (StatLab) for hemoparasite microscopic detection. At necropsy, we observed moderately decreased pectoral muscling and scant adipose tissue stores. The esophagus, crop, gizzard, and proventriculus contained green, fetid, turbid liquid. A large number of ascarids were present in the lumen of the proventriculus, gizzard, duodenum, and jejunum, and a small number were present in the lumen of the trachea, esophagus, and crop. Ascarids were tan and approximately 3.5–4.5 cm in length. The liver was moderately enlarged and green-tinged, and there were small, firm, tan scattered nodules throughout the hepatic lobes. A focal, coiled adult nematode was embedded in the liver parenchyma (Figure 1a). The heart was mildly distended and flaccid. Tissue sections from the eye, cerebrum, cerebellum, trachea, heart, lungs, liver, spleen, kidneys, pectoral muscles, esophagus, crop, proventriculus, ventriculus, and intestines were collected and fixed in 10% buffered formalin (pH 7.2) for 24–48 h. Swabs from the heart, trachea, liver, and spleen were cultured onto 5% sheep blood agar (Hardy Diagnostics, Santa María, CA, USA) and MacConkey agar plates (Hardy) and incubated with 7% CO_2_ at 37 °C for 48 h. Swabs of the liver and the large intestine were inoculated into tetrathionate broth base (Hardy) for *Salmonella* sp. isolation. An oropharyngeal swab and cloacal swab were tested for the avian IAV matrix gene by RT-qPCR. The liver tissue was tested for *Chlamydia psittaci* by qPCR. Ascarids from the gastrointestinal tract and trachea were collected for morphological assessment using direct microscopy. Genotypic identification of the parasite was performed with PCR of an 815 bp segment of the 18S rRNA gene using primers Nem_18S_F (CGCGAATRGCTCATTACAACAGC) and Nem_18S_R (GGGCGGTATCTGATCGCC) using the methodology previously described [8]. The PCR amplicon was evaluated on an Agilent TapeStation for a ~815 bp band. Amplicon purification was performed using ExoSAP-It™ (Applied Biosystems, Waltham, MA, USA) according to manufacturer instructions. Sanger sequencing was performed by Elim Biopharmaceuticals (Hayward, CA, USA). The sequence was analyzed using Geneious Prime (2026.1) and NCBI BLAST (2.17.0).

**Figure 2:**
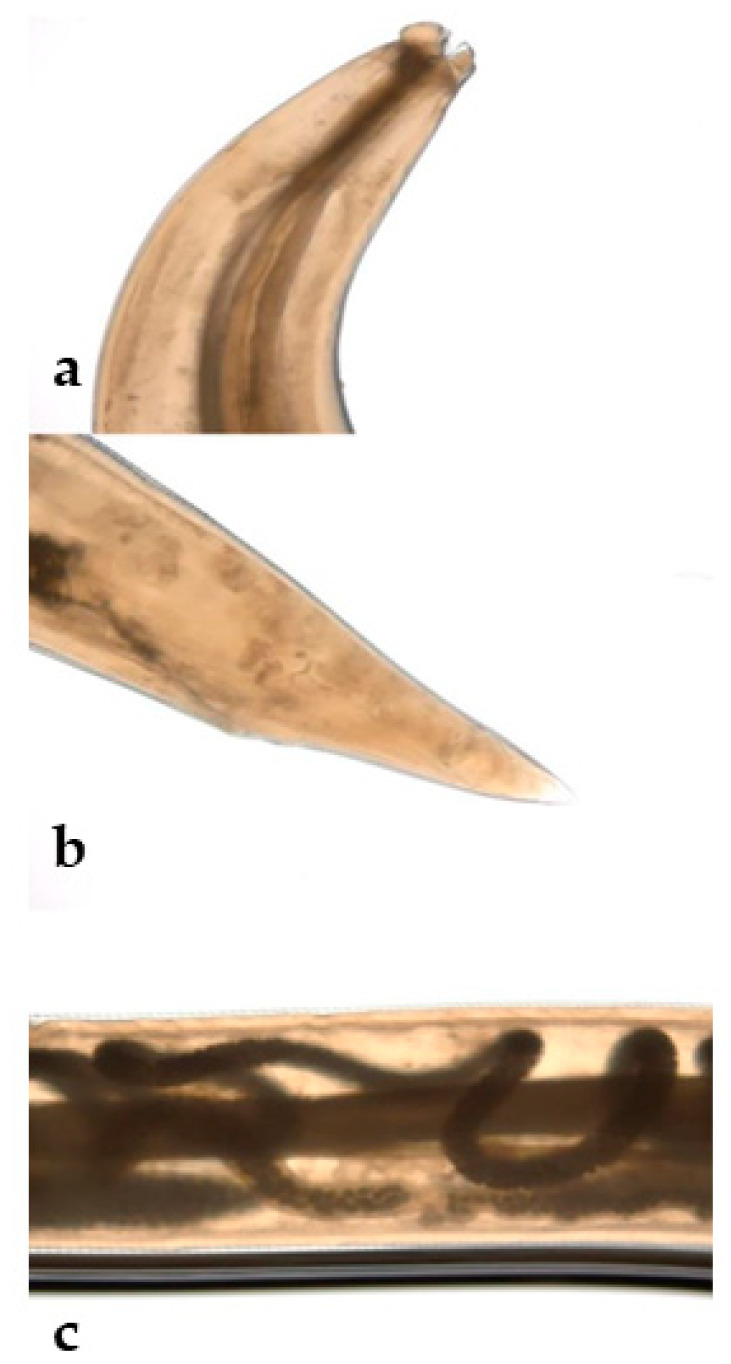
(**a**–**c**) Microscopic images of *Ascaridia* sp. from the intestine of the studied domestic pigeon. (**a**) Lateral view of the cephalic end with trilobed lips. (**b**) Conical tail of the female worm. (**c**) The cuticle with the transverse striations and uteri with ova present.

**Figure 3:**
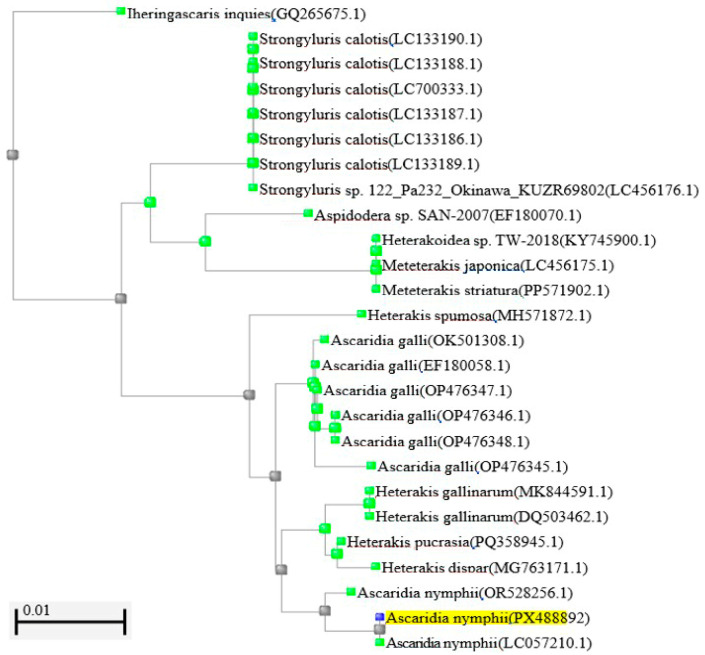
Phylogenetic analysis of the 18s rRNA gene from our case (GenBank accession PX488892) highlighted in yellow, sharing 100% identity with *Ascaridia nymphii* from GenBank accession LC057210.1.

No bacterial pathogens were isolated from aerobic cultures. Avian IAV and *C. psittaci* were not detected by RT-qPCR and qPCR tests, respectively. No hemoparasites were detected in Diff-Quick-stained blood smears. The serum sample was seronegative for avian IAV, avian PMV-1, 2, 3, MG, MS, *S*. Pullorum, and *S*. Gallinarum. On the histopathology, multifocally effacing more than 50% of the liver were coalescing, variably sized granulomas. Granulomas were up to 0.5 cm in diameter with necrotic centers delineated by multinucleated giant cells, fibrous connective tissue, fibroblasts, lymphocytes, and eosinophils (Figure 1b). Within the granulomas were cross-sections of larval nematodes and colonies of bacterial cocci. Multifocal sub-acute migration tracts composed of circumscribed hemorrhage, necrosis, fibrin accumulation, and modest numbers of eosinophils with a narrow rim of fibrocytes were distributed randomly throughout the liver (Figure 1b). There was also marked proliferation of bile ducts and fibroplasia (ductular reaction) throughout the liver. Large numbers of adult and larval nematodes filled the distended duodenal and jejunal lumen. Adult nematodes had a serrated cuticle, with lateral alae and reproductive tracts that often contained thick-shelled eggs [15]. In the ventriculus, the koilin was markedly fibrillated and eroded, and numerous, similar adult nematodes were embedded within the koilin, adjacent to the myriad ova and colonies of the rod-shaped bacteria. The ventricular mucosa was multifocally necrotic and ulcerated with moderate eosinophilic infiltrates (Figure 1c). In addition, there was severe granulocytic enteritis, crypt dilation with necrotic debris, and accumulation of large quantities of cellular debris on the intestinal mucosa. A longitudinal section of the adult nematodes was within the tracheal lumen. No larval nematodes were noted in the liver and lungs. In this case, the nematode microscopic morphologic features included a pseudocoelom containing lateral chords and coelomyarian musculature; a cuticle ornamented with lateral alae; a cephalic end with trilobed lips and without an interlabia (Figure 2a); the female worm had a conical tail that measured 1.27 mm (Figure 2b) and a club-shaped esophagus; and uteri filled with eggs (Figure 2c), providing the diagnosis of *Ascaridia* sp. The genotypic characterization of the *Ascaridia* sp. from our case (GenBank database accession PX488893) shared 100% identity with *A. nymphii* from the intestinal tract of the cockatiel from Japan in 2015 (GenBank database accession LC057210.1) based on 18S rRNA PCR and sequencing alignment (Figure 3).

## 3. Discussion

In our case, we found an unusually high *A. nymphii* burden that affected the upper and lower digestive tracts and trachea, causing hepatic granulomas and migration routes in a domestic pigeon. We combined morphologic and 18S rRNA PCR and sequencing for the identification of *A. nymphii*, considering that additional loci identification would provide stronger phylogenetic species confirmation.

We found no previous reports of accidental *A. nymphii* infection causing gastrointestinal impaction and hepatic migration affecting avian species in a search in Google Scholar, PubMed, SciELO, CAB Direct, ResearchGate, Web of Science, and Scopus using the terms “*Ascaridia nymphii*”, “accidental infection”, “hepatic migration”, “avian”, “birds” and “pigeon”. Based on the postmortem diagnostic tests performed, no concurrent viral, bacterial, fungal, or other parasitic agents were present in the case of this pigeon.

To our knowledge, accidental *A. nymphii* infection causing gastrointestinal impaction and hepatic migration has not been previously reported in domestic pigeons. We conjecture that the lack of bacterial growth despite the presence of the localized cocci colonies noted on the hepatic microscopic evaluation could be associated with the chronicity of the lesions or possible technical mistakes on the hepatic targeted area of the swab and culture. The life cycle of ascarids is direct via the fecal-oral route. In short, unembryonated eggs are passed in the feces and subsequently embryonate into larvated eggs in 14–30 days in the environment. After ingestion by an avian host, the eggs hatch and release the second larval stage (L2), and then they migrate into the small intestinal mucosa and molt to the third larval stage (L3), after which they finally return to the lumen to complete molting to the adult stage [4,6,9,10]. Accidental infections, which occur when a non-typical host acquires an ascarid infection, should be differentiated from aberrant migrations, which involve the migration of a larval stage to an area where it does not typically migrate [6]. Hepatic and pulmonary migration of *Ascaridia* sp. has been previously reported in columbiform, galliform, and psittaciform birds [7,10,11,16,17,18,19]. In pigeons, *A. columbae* L2 often migrate to hepatic parenchyma via the portal vein and subsequently to the lungs, where ascarids are coughed up, swallowed, and then re-enter the gastrointestinal tract. Larval migration induces granulomatous inflammation under natural and experimental conditions [7,9,10,11]. In the table-egg industry, *A. galli* adult worms in chicken egg layers have been reported to migrate from the cloaca to the shell gland of the oviduct, where they can subsequently be incorporated into the egg, which leads to public health concerns and egg-quality issues [16,20].

*Ascaridia hermaphrodita* and *Ascaridia dissimilis* larvae have been described in cases of hepatic migration in a naturally infected blue-fronted Amazon parrot (*Amazona aestiva*) [18] and experimentally inoculated white turkey poults, respectively [19]. In our case, other gastrointestinal roundworm endoparasitisms different from *A. columbae* and *A. galli* infections such as *Capillaria* spp., *Hadjelia truncata*, *Synhimantus* (*Dispharynx*) *nasuta*, and *Tetrameres* spp. infections were ruled out based on the affected digestive tract location, size, morphological features, and genotypic characterization of the parasites found in the studied domestic pigeon [4,6,9].

Both benzimidazoles (e.g., fenbendazole and albendazole), which are the only anthelmintic class approved for commercial poultry in the United States and the European Union for the removal of adult and larval ascarid stages [20], and imidazothiazoles (e.g., levamisole and pyrantel), which are also recommended for ascarid control in avian species not utilized in food production [4,20], represent good options for ascarid treatment and control in the remaining domestic pigeons of the studied case. In this case, a multidirectional approach to treat, control, and prevent further parasitic infections in the flock is recommended and should include the rotation of anthelmintic drugs to reduce the occurrence of anthelmintic resistance, caused by repeated treatment with the same drug class [20]; reducing environmentally resistant ascarid eggs and accumulation of potentially viable eggs with infective L2 addressed by management strategies such as regular removal of organic matter; avoiding the entrance of apparently healthy pigeons without quarantine; cleaning and disinfecting the loft and limiting contact of the domestic pigeons with free-ranging birds; and biannual monitoring of parasite loads in the loft by diagnostic analysis of the periodical fecal samples [20].

## 4. Conclusions

We reported the *A. nymphii* infection in a domestic pigeon based on the gross and microscopic postmortem findings and nematode microscopic identification together with PCR and sequencing studies, without concurrent infections. While *A. columbae* and *A. galli* infections are the most prevalent ascarid species parasitizing Columbiformes, with and without lung-liver migration, respectively, *A. nymphii* infection and its life cycle have not been previously described in pigeons in the current literature. Here, we contribute to the understanding of *A. nymphii* infection in an avian species not previously reported as a potential host parasitized by this barely reported ascarid species. Follow-up studies may be warranted to investigate the pathogenicity of *A. nymphii* in Columbiformes and determine the susceptibility of other avian host species.

## Data Availability

Data is contained within the article.
